# Optimization of Dissolution Compartments in a Biorelevant Dissolution Apparatus Golem v2, Supported by Multivariate Analysis

**DOI:** 10.3390/molecules22122042

**Published:** 2017-11-23

**Authors:** Ivan Stupák, Sylvie Pavloková, Jakub Vysloužil, Jiří Dohnal, Martin Čulen

**Affiliations:** 1 Department of Chemical Drugs, Faculty of Pharmacy, University of Veterinary and Pharmaceutical Sciences Brno, Palackeho 1, 612 42 Brno, Czech Republic; ivan.stupak@gmail.com; 2 Department of Pharmaceutics, Faculty of Pharmacy, University of Veterinary and Pharmaceutical Sciences Brno, Palackeho 1, 612 42 Brno, Czech Republic; pavlokovas2@vfu.cz (S.P.); jakub.vyslouzil@gmail.com (J.V.); 3 Department of Social Pharmacy, Faculty of Pharmacy, University of Veterinary and Pharmaceutical Sciences Brno, Palackeho 1, 612 42 Brno, Czech Republic; Dohnalj@vfu.cz; 4 Central Laboratories, Faculty of Pharmacy, University of Veterinary and Pharmaceutical Sciences Brno, Palackeho 1, 612 42 Brno, Czech Republic

**Keywords:** dissolution, biorelevant, Golem, caffeine, multivariate data analysis

## Abstract

Biorelevant dissolution instruments represent an important tool for pharmaceutical research and development. These instruments are designed to simulate the dissolution of drug formulations in conditions most closely mimicking the gastrointestinal tract. In this work, we focused on the optimization of dissolution compartments/vessels for an updated version of the biorelevant dissolution apparatus—Golem v2. We designed eight compartments of uniform size but different inner geometry. The dissolution performance of the compartments was tested using immediate release caffeine tablets and evaluated by standard statistical methods and principal component analysis. Based on two phases of dissolution testing (using 250 and 100 mL of dissolution medium), we selected two compartment types yielding the highest measurement reproducibility. We also confirmed a statistically ssignificant effect of agitation rate and dissolution volume on the extent of drug dissolved and measurement reproducibility.

## 1. Introduction

Due to a noticeable portion of generic drug products in the present pharmaceutical market, more attention is paid to the topics of generic development and bioequivalence studies. Introduction of the Biopharmaceutical Classification System (BCS) enabled the substitution of pharmacokinetic (PK) in vivo studies with dissolution tests in the case of products with well soluble and permeable active ingredients [1,2]. For other drug classes, biorelevant dissolution tests can be utilized to obtain a prediction of in vivo dissolution performance of a drug product, which provides a valuable guidance before commencing a PK study. Dissolution data can also be used to develop an in vitro in vivo correlation which can serve as a waiver of in vivo testing, specifically for BCS Class I drugs (high solubility, high permeability) and the BCS Class III (high solubility, low permeability) [3,4,5]. Understanding the physiology of the human gastrointestinal tract remains essential for the successful development of a biorelevant dissolution setup [6]. Unlike conventional dissolution methods, these should address, more closely, the physiological aspects of the human gastrointestinal tract (GIT) [7,8]. The biorelevant dissolution systems, described to date, vary in their complexity and the technical solutions used for utilizing peristaltic movement, pH changes, diverse fluid composition, volume, transit, secretion, and absorption. The most advanced systems for simulation of gastric conditions include the Dynamic Gastric Model (DGM) [9,10], the Human Gastric Simulator (HGS) [11], and the TNO Gastro-Intestinal Model with an advanced gastric compartment, named as “TIMagc” [12]. These apparatuses feature a very close simulation of gastric stress forces, along with gastric secretions. Several other instruments enable also the simulation of conditions in the small intestine. One of the most complex models, the TIM-1, enables a full simulation of the gastro-intestinal (GI) transit, from the stomach through three separate parts of the small intestine, while being able to maintain the physiological composition of the artificial chyme [13,14]. Novel instruments include the Engineered Stomach and Small Intestinal System (ESIN), with differential gastric emptying of solids and liquids, and the possibility of a progressive meal intake [15]. A close simulation of peristaltic contractions in the small intestine, was achieved in the Intestine Model for Simulating the Peristaltic Action (IMSPA), by using a hollow silicon rubber tube surrounded by four constriction mechanisms [16]. All the above-mentioned bio/physiologically relevant instruments differ in the technical solutions applied and the extent of simulated conditions, however their utilization follows one goal, which is the most accurate prediction of the in vivo dissolution performance.

**Figure 1:**
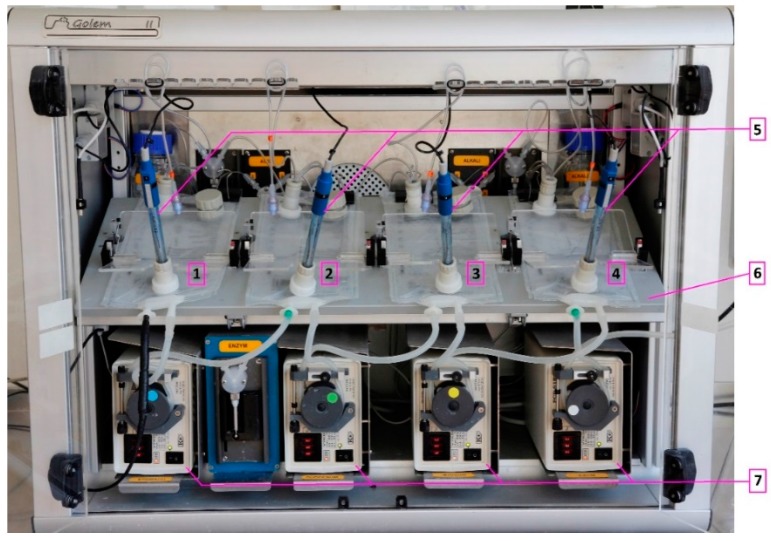
Golem v2 apparatus photographed from the front view: (1) stomach, (2) duodenum, (3) jejunum, (4) ileum, (5) pH probes, (6) heating platform, and (7) peristaltic pumps.

To provide a means of highly biorelevant in vitro dissolution testing, our team has introduced the ‘Golem’ apparatus, a dynamic four-compartmental dissolution instrument which simulates chyme transit and biorelevant conditions in the stomach and three parts of small intestine—duodenum, jejunum, ileum. A detailed description of the apparatus is provided in a previous work [17]. In a fully biorelevant setup, the apparatus design allows for simulation of physiological conditions with the possibility of adjusting all vital parameters such as pH, volumes, transit times, temperature, agitation/peristaltic rate, and enzyme secretion during the testing run. Recently, the design of the Golem apparatus (Figure 1) was revised, introducing several minor technical updates, including an electrically-driven agitation/peristaltic system with autonomous mixing for each compartment. As one of the most important components of any biorelevant dissolution instrument is its dissolution vessel(s), we also proposed a new design of the dissolution compartments. The compartments in the Golem apparatus are created from standard plastic medical infusion bags, which allowed their simple modification into different shapes. Our preliminary experiments demonstrated a strong influence of the modified inner geometrics on the dissolution profiles and the measurement reproducibility. Therefore, prior to future biorelevant dissolution testing on Golem v2, this study was conducted with the primary aim to test various types of modified compartments, differing in inner geometry, and to select the compartment type yielding the highest reproducibility. As secondary aims, we assessed the effect of agitation rate, which can be changed on the scale from 0 to 7 lifts (revelations) of the paddle per minute (LPM), and also investigated the effect of two different dissolution medium volumes—250 mL and 100 mL.

## 2. Results and Discussion

### 2.1. Experimental Design

**Figure 2:**
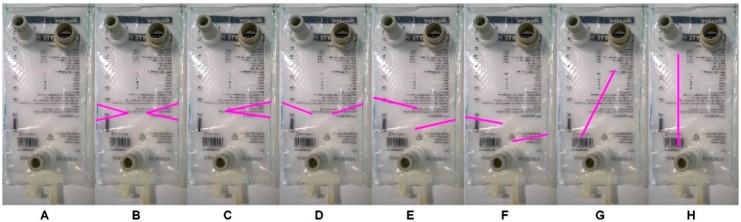
Compartment designs. The intravenous bags were constricted at specific positions by welding, as highlighted by magenta lines. Letters denominate the individual compartment types (**A**–**H**).

Seven different compartment geometries were created by intersecting the inner space of the infusion bags. Unmodified version (type A) served as a reference compartment (Figure 2). The dissolution tests were performed using immediate release caffeine tablets, at two agitation (peristaltic) rates—high (7 LPM) and low (3 LPM)—in phosphate buffer (pH 6.8) dissolution medium. In Phase 1, we tested the dissolution in 250 mL of medium, representing the gastric content after administration of oral dosage form in fasted state conditions. Based on the evaluation of reproducibility, the four most robust compartment types (B, C, D, F), plus reference (type A), were selected for further testing in Phase 2, using only 100 mL of medium. The lower dissolution volume was chosen to represent momentarily conditions encountered during GI transit simulation [6]. Also, we expected that lower dissolution volume would increase measurement variability and thus tighten the robustness criteria. After selection in Phase 2, the dissolution profiles of the 2 most robust compartment types, B and C (3 and 7 LPM agitation; 100 mL volume) were compared with a standard USP 2 dissolution test (50 rotations per minute (RPM); 500 mL volume).

#### 2.1.1. Compartment Type Analysis—Phase 1

**Figure 3:**
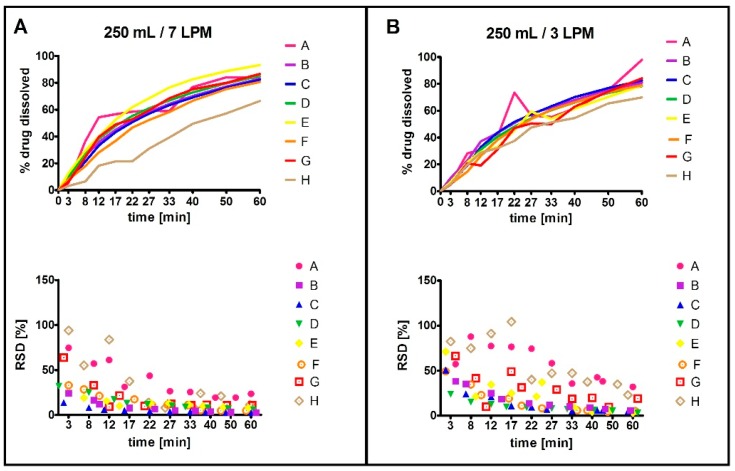
Dissolution data from Phase 1 (250 mL), tested at 7 LPM (**A**) and 3 LPM (**B**) agitation rate. The bottom graphs show the individual RSD values for each time point.

**Figure 4:**
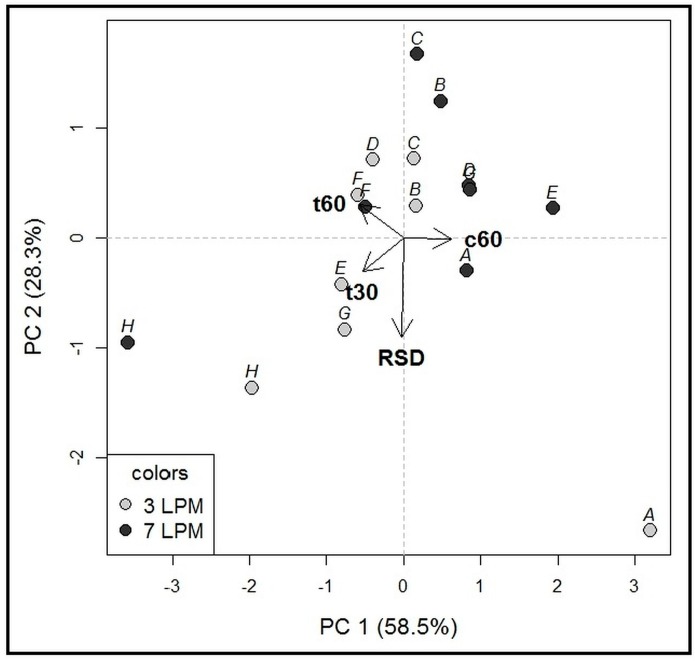
PCA biplot for Phase 1 (250 mL volume). Objects included in the model: eight dissolution compartment types (designated A-H), two agitation rates—7 and 3 LPM. Variables included in the model: t30, t60, c60, and median RSD. The first two principal components (PC1, PC2) explain 86.8% of the total variability, which is sufficient [18]. The PC1 was predominantly associated with the c60 (positive loadings), while the PC2 was mainly influenced by median RSD (negative loadings). Therefore, the measurement precision did not correlate with the amount of drug dissolved at the final time point (c60). Object clustering according to the agitation rate, especially in the direction of the vectors c60 and t30, demonstrates that higher agitation rate resulted in more rapid dissolution. Higher agitation rate was also associated with lower median RSD values, demonstrated by different positioning of 3 LPM versus 7 LPM measurements. The measurement precision increased in the opposite direction to the median RSD vector. The highest robustness/reproducibility was obtained for the compartment types B, C, D and F.

The dissolution performance in individual compartment types, each representing different inner geometry, was assessed using four values: the time required to reach 30% and 60% of drug dissolved (designated as t30, t60, respectively), the amount of drug dissolved at last time point, i.e., 60 min (designated as c60), and median relative standard deviation (RSD) for each compartment (Figure 3). An individual RSD (standard deviation/mean × 100) of multiple measurement repetitions was first calculated for each individual sampling time point (e.g., at 3 min). Next, the median of the individual RSDs calculated for the individual sampling time points was obtained, referred to as the median RSD. Statistical analysis revealed a difference between dissolution profiles among five of the eight compartments (C, D, E, G, H; paired *t*-test, *p* < 0.05). Importantly, we observed a statistically significant difference between the median RSD values, i.e., reproducibility/robustness, of the individual compartment types (analysis of variance (ANOVA), *p* < 0.05). The results of statistical testing were in line with the PCA output (Figure 4). Four compartments with the best reproducibility (B, C, D, F) were selected for Phase 2 testing.

#### 2.1.2. Compartment Type Analysis—Phase 2

**Figure 5:**
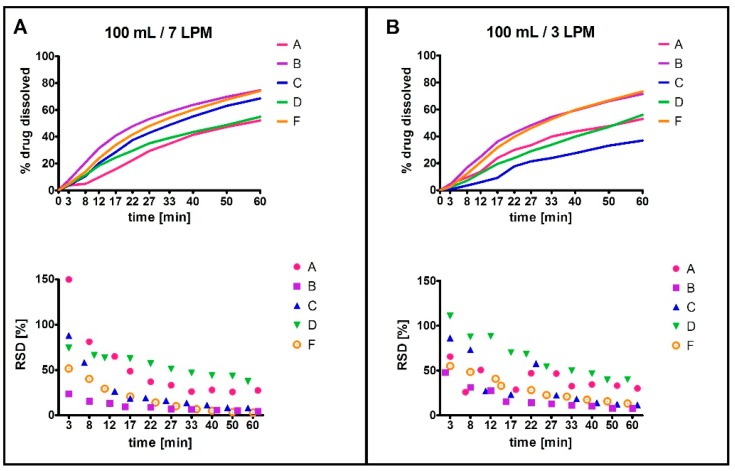
Dissolution data from Phase 2 (100 mL), tested at 7 LPM (**A**) and 3 LPM (**B**) agitation rate. The bottom graphs show the individual RSD values for each time point.

**Figure 6:**
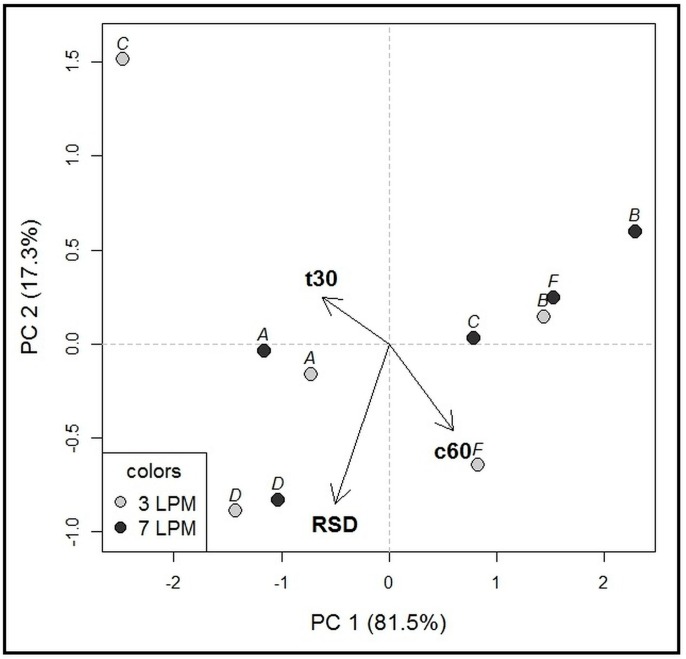
PCA biplot for Phase 2 (100 mL volume). Objects included in the model: five dissolution compartment types (A, B, C, D, F), two agitation rates—7 and 3 LPM. Variables included in the model: t30, c60, median RSD. Higher agitation rate was associated with lower median RSD values (different positioning of 3 LPM and 7 LPM measurements). Highest robustness was obtained for the compartment types B and C.

Testing at 100 mL dissolution medium volume showed statistically significant difference between the dissolution profiles of all four selected compartment types (Figure 5), plus the reference type A (paired *t*-test, *p* < 0.05). Only t30 and c60 values were evaluated, as t60 was not reached for all compartments. The compartments demonstrated different robustness (comparison of median RSD value; ANOVA *p* < 0.001). The results of the statistical analysis were in agreement with PCA output (Figure 6). Based on median RSD value and PCA, compartment types B and C were chosen as the most robust.

#### 2.1.3. Influence of Agitation Rate and Dissolution Volumes

**Figure 7:**
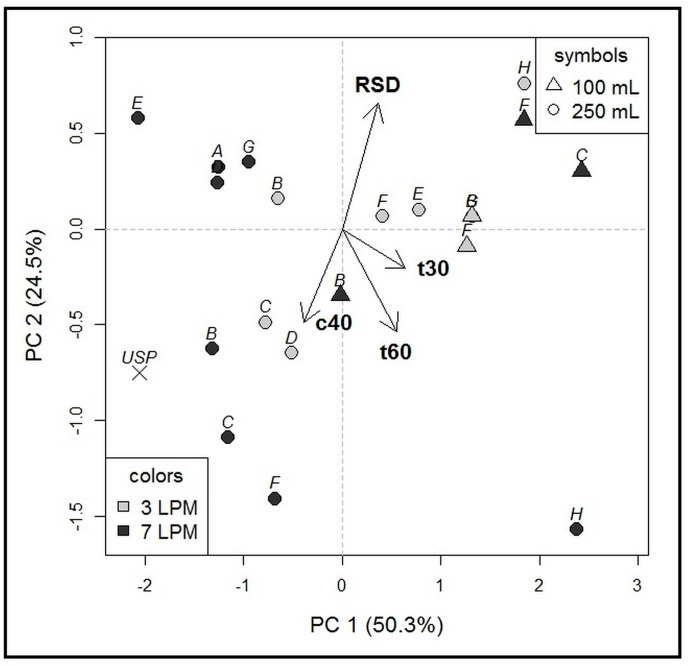
Complex PCA biplot for merged data from Phase 1 and Phase 2. The multivariant analysis was used to assess the general effect of agitation rate and volume on measurement reproducibility and dissolution at in Golem v2. Objects included in the model: eight dissolution compartment types (designated A–H), two agitation rates—7 and 3 LPM, two volumes—100 and 250 mL. Variables included in the model: t30, t60, median RSD, and amount of drug dissolved at 40 min (c40). Clustering of objects according to dissolution volume was observed. USP 2 dissolution results were compared with compartment B and C, where higher similarity with USP 2 was observed for 250 mL.

The statistical analysis confirmed that agitation rate influenced dissolution, with higher rates resulting in higher amount of drug dissolved. This was observed for experiments with both 250 and 100 mL of dissolution volume, although for 100 mL the relationship was confirmed only by *t*-test (250 mL—ANOVA *p* < 0.01, paired *t*-test *p* < 0.05; 100 mL—ANOVA *p* > 0.05, paired *t*-test *p* < 0.05). The statistical results were in line with the PCA output (Figure 7). These observations and the fact that the agitation rate can be further lowered to 1 LPM indicate that peristaltic simulation in Golem v2 provides a key parameter for the control of dissolution rate. For the compartment types B and C, the latter produced the highest difference between 7 and 3 LPM (difference of 27 min, for t30), indicating that type C compartment may be preferred if a wider range of peristaltic conditions is to be simulated. Based on the median RSD, higher measurement reproducibility was observed with the higher dissolution volume (250 mL) and higher agitation rate (7 LPM; ANOVA *p* < 0.05). Although, the 100 mL of dissolution medium represented a sufficient sink condition for caffeine (>5 × volume of saturated solution; aqueous solubility 32–39 mg/mL, at 37 °C), we observed a significantly higher dissolution in 250 mL volume (ANOVA mixed models approach to within-subject factors, *p* < 0.001, and paired *t*-test for all compared pairs, *p* < 0.05), confirmed also by PCA biplot (Figure 7) [19]. The effect of dissolution volume was statistically significant for all compartments (ANOVA mixed models approach to within-subject factors, *p* < 0.001, and paired *t*-test for all compared pairs, *p* < 0.05). This underlines the importance of correct simulation of physiologically relevant volumes present in separate parts of GIT. Of note, the volume of liquid in the stomach and the small intestine in the fasted state was reported to be around 30 mL and 100 mL, respectively, before the administration of dosage form and water [6].

#### 2.1.4. USP 2 Comparison

**Figure 8:**
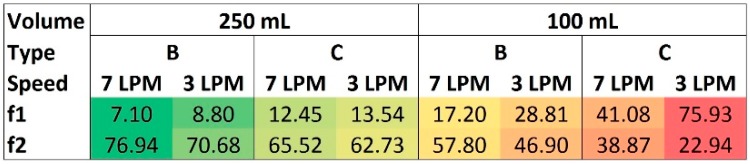
Difference (f1) and similarity (f2) factor analysis for comparison of compartment types B and C with the USP 2 dissolution test. Profiles were similar if f1 < 15 and f2 > 50.

As the final step, the dissolution profiles for the most robust compartment types, B and C, measured with 250 and 100 mL of medium were compared to USP 2 dissolution test, measured with 500 mL at 50 rpm, as a standard setup. The dissolution profiles from Golem v2 at 250 mL, corresponded to USP2, as assessed by difference and similarity factor analysis (f1 and f2, see Figure 8), and supported by PCA output (Figure 7). However, the Golem v2 dissolution profiles measured at 100 mL, showed discrepancy from the USP2 dissolution profiles. The difference was more pronounced with compartment type C and lower agitation rates in Golem v2. These data clearly demonstrate the need for a specialized biorelevant dissolution devices like the Golem apparatus, when simulating physiologically relevant low chyme volumes and lower peristalsis [20].

## 3. Materials and Methods 

### 3.1. Tablets

Immediate release caffeine tablets were prepared by direct compressing (Korsch type EK 0, Korsch Pressen, Berlin, Germany) of tableting mixture (caffeine anhydrous 12.5% *w*/*w*, Kulich Pharma, s.r.o., Hradec Kralove, Czech Republic; magnesium stearate 2.0% *w*/*w*, Peter Greven, Bad Munstereifel, Germany; Avicel PH 102 42.75% *w*/*w*, FMC International, Cork, Ireland; Pharmatose DCL 42.75% *w*/*w*, DFE Pharma, Goch, Germany) into 7 mm flat tablets, weighting 150 mg ± 0.2%. The tablets were tested and conformed to Ph. Eur. 8 requirements for weight uniformity (150.3 ± 1.0 mm; analytical balance KERN 870-13, KERN &amp; Sohn Gmgh, Balingen, Germany), hardness (73.7 ± 3.0 N; C50 Tablet Hardness &amp; Compression Tester, Engineering Systems, Nottingham, Great Britain), friability (0.41%; ERWEKA TAR 10, Erweka, Heusenstamm, Germany), disintegration test (10 min and 3 s; ERWEKA ZT4, Erweka, Heusenstamm, Germany) and drug content by spectrophotometric methods at 273 nm (19.43 ± 0.24 mg; Lambda 25, UV/Vis spectrometer, Perkin Elmer, Wellesley, MA, USA) in pH 6.8 phosphate buffer [21].

### 3.2. Golem v2 Dissolution Compartments

Baxter Viaflo (Baxter Healthcare Ltd., Thetford, UK) two layers (polyolefin/polyamide) non-PVC intravenous medical bags were modified by welding to provide a specific inner geometry. Eight compartment designs of uniform size (12 cm width × 24 cm height) but various inner geometries (Figure 2) were manufactured in collaboration with Institute of Organic Chemistry and Biochemistry (Czech Academy of Sciences, Prague, Czech Republic).

### 3.3. Dissolution Testing in Golem v2 Apparatus

Prior to the experiments, the dissolution medium (pH 6.8 phosphate buffer) was pre-heated to 37.0 ± 0.5 °C. According to the test conditions, 250 mL or 100 mL of dissolution medium was used in combination with agitation rate of either 3 or 7 LPM. Samples were manually collected at 3, 8, 12, 17, 22, 27, 33, 40, 50 and 60 min after the tablet administration (time points were taken from previous automated Golem analysis). All experiments were performed with five replicates.

### 3.4. HPLC Analysis

The samples from the Golem v2 apparatus were filtered through 0.2 μm nylon syringe filter and the caffeine dissolved using a high-performance liquid chromatography (HPLC). The HPLC system Agilent 1260 Infinity (Agilent Technologies, Waldbronn, Germany) consisted of a quaternary pump, degasser, autosampler, column oven, and diode array detector. The chromatographic separation was performed in reversed phase, using Supelcosil ABZ + Plus column (150 mm × 4.6 mm × 3 μm, Sigma-Aldrich, Bellafonte, PA, USA). Mobile phase composition was isocratic, consisting of methanol and 50 mM acetate buffer (20:80, *v*/*v*), adjusted to pH 4. The flow rate was kept at 1.5 mL/min. The injection volume was set to 10 μL and the column temperature was maintained at 40.0 °C. The spectra were recorded from 190 to 400 nm, while chromatogram was acquired at 294 nm. Each analysis was performed in triplicate.

### 3.5. USP Dissolution Apparatus 2 Test

The USP 2 dissolution test was performed using Sotax AT 7, on-line system (Donau Lab, Zurich, Switzerland). The dissolution medium, pH 6.8 phosphate buffer of 500 mL volume was kept at 37.0 ± 0.5 °C and stirred at 50 RPM. Quantification of dissolved caffeine was performed using coupled UV spectrophotometer Lambda 25 (Perkin Elmer, St. Louis, MO, USA) at 273 nm.

### 3.6. Statistical Analysis

The aim of the statistical analysis was to determine the effect of dissolution compartments, dissolution medium volume, and agitation rate on the resulting dissolution characteristics. It was also used to ease the identification of compartments with the highest robustness. Principal component analysis (PCA) was used to describe the multivariate dependence structure between variables. Significance testing using ANOVA and paired *t*-test were employed as standard statistical methods. The statistical analysis was performed separately for the Phase 1 and Phase 2 of the experiment. Data analysis was performed by means of QC.Expert software 3.2, and the R software, version 3.2.2. [22]. Comparison of Golem v2 and USP 2 dissolution profiles were performed via difference (f1) and similarity (f2) factor calculation [23].

## 4. Conclusions

The primary aim of this study was to test different designs of the dissolution compartments, applicable in the Golem v2 apparatus. Based on a robustness criterion, we selected two compartment types with the highest reproducibility, as supported both by the standard and multivariate statistical analysis. In addition, we also confirmed the discriminatory effect of agitation rate and dissolution volume on the dissolution of immediate release caffeine tablets. Testing with 250 mL of medium volume and higher agitation rate (7 LPM) produced dissolution curves similar to the standard USP 2 test. However, extending the simulation conditions to lower volume (100 mL) and lower agitation (3 LPM) produced significantly different results to the standard USP 2 test, which underlines the capacity of Golem v2 for simulation of more physiologically relevant conditions. The selected compartments and conclusions of this study will be further implemented for simulations of the full GI transit.
